# Eight-year operation status and data analysis of the first human milk bank in East China

**DOI:** 10.1186/s13006-022-00502-8

**Published:** 2022-09-01

**Authors:** Hu Xiaoshan, Chu Xue, Zhang Jun, Liu Feng, Chen Xiaohui, Yu Zhangbin, Han Shuping

**Affiliations:** Department of Pediatrics, Maternity Hospital Affiliated to Nanjing Medical University/Nanjing Maternal and Child Health Hospital, 123 Tianfeixiang,Mochou Road, Nanjing, China

**Keywords:** Human milk bank (HMB), COVID-19, Donor human milk, Breast milk, Newborn, Recipients, Preterm birth

## Abstract

**Background:**

Human milk banks (HMBs) are essential facilities for the selection, collection, testing, transportation,storage, and distribution of DHM for special medical needs. The aim of this analysis was to analyze the operation status and data over the last 8 years of operation of the first human milk bank (HMB) in East China.

**Methods:**

Data related to the costs, donors, donation, pasteurization, and recipients were extracted from the web-based electronic monitoring system of the HMB for the period August 1, 2013 to July 31, 2021.

**Results:**

Over the 8 years of operation, 1,555 qualified donors donated 7,396.5L of qualified milk at a cost of ¥1.94 million($306,051), with the average cost per liter of donor human milk being ¥262.3($41.4). The donors were between 25 and 30 years of age, and the majority (80.1%) were primipara. All the donated milk was pasteurized and subjected to bacteriological tests before and after pasteurization: 95.4% passed the pre-pasteurization tests, and 96.3% passed the post-pasteurization tests. A total of 9,207 newborns received 5,775.2L of pasteurized donor milk. The main reason for the prescription of donor human milk was preterm birth. As a result of continuous quality improvements, January 2016 witnessed a significant increase in the volume of qualified DHM and the number of qualified donors. However, in 2020, as a result of the restrictions related to the COVID-19 pandemic, the volume of qualified DHM and the number of qualified donors decreased.

**Conclusions:**

Over its 8 years of operation, our HMB has made steady quality improvements in its screening and information processes. Continuous quality improvement is on ongoing need, along with recruiting more qualified donors and collecting donor human milk for vulnerable newborns.

## Background

Breast milk alone is the optimal source of nutrition for newborns for the first 6 months after birth [1]. Compared to formula, both breastfeeding and donor human milk (DHM) have immense advantages, as they are associated with reduced rates of chronic lung disease, necrotizing enterocolitis, feeding intolerance, nosocomial infection, retinopathy of prematurity, and mortality in premature infants [2–4]. Therefore, donor DHM is preferred to formula when maternal milk is absent or insufficient, as recommended by the World Health Organization [5]. Although the properties of DHM are affected by the processes of collecting, processing, and storing, it is still superior to formula in terms of nutritional composition and biological value [6, 7].

Human milk banks (HMBs) are essential facilities for the selection, collection, testing, transportation, storage, and distribution of DHM for special medical needs. The first HBM was established in 1909 in Vienna, and since then HMBs have been set up in Europe and in the United States and have been in operation for more than 100 years [8]. The first HMB in China was opened in March 2013 in Guangzhou [9], and as of 2019, China had 19 HMBs [10]. Our HMB is the first and largest HMB in East China. It was founded in August 2013 according to the standards and guidelines of the Human Milk Banking Association of North America, and has been in operation for 8 years. Over its 8 years of operation, a total of 7,396.5 L of qualified DHM has been collected, and 9,207 newborns have received DHM from our bank. We have made continuous quality improvements over these 8 years, and the purpose of this study is to describe these changes and the operation status of our HMB over the 8 years,and provide some recommendations to other HMBs operating in China.

## Methods

### Data resources and research variables

Data from August 2013 to July 2021 were extracted from the computerized information management system of our HMB. The extracted donor data included age, education, residence address, occupation, number of children, number of donations, etc. Additionally, the results of bacteriological tests done before and after pasteurization were obtained. The extracted recipient data included gestational age, birth weight, mode of childbirth, number of days that DHM was used, etc.

### Donation process

Prior to January 2016, donors who passed the health screening, serological test(including hepatitis B virus(HBV),hepatitis C virus(HCV), Human Immunodeficiency Virus(HIV), syphilis and cytomegalovirus(CMV)), and their DHM passed the bacteriological tests (including bacterial species and count of bacterial colonies,both pre- and post-pasteurization) at the time of the first donation were considered to be qualified and allowed to donate in the future. Then their DHM was used without pre- or post-pasteurization bacteriological tests. After the continuous quality improvements [11] starting in January 2016, donors are also eligible if they pass the health screening, serological test, and and their DHM passed the bacteriological tests(both pre- and post-pasteurization tests) at the time of the first donation. But these qualified donors’ DHM at the same day was first pooled and then pasteurized. In the early stage of quality improvement, bacteriological test was conducted before and after the pasteurization of each batch every day. It was subsequently found that our milk extraction process and pasteurization process were reliable if the batches passed most of the tests, and thereafter, bacteriological test was conducted every 10 days. These batches was discarded before pasteurization if failing the pre-pasteurization bacteriological test, and also discarded after pasteurization if failing the post-pasteurization bacteriological test.

**Fig. 1:**
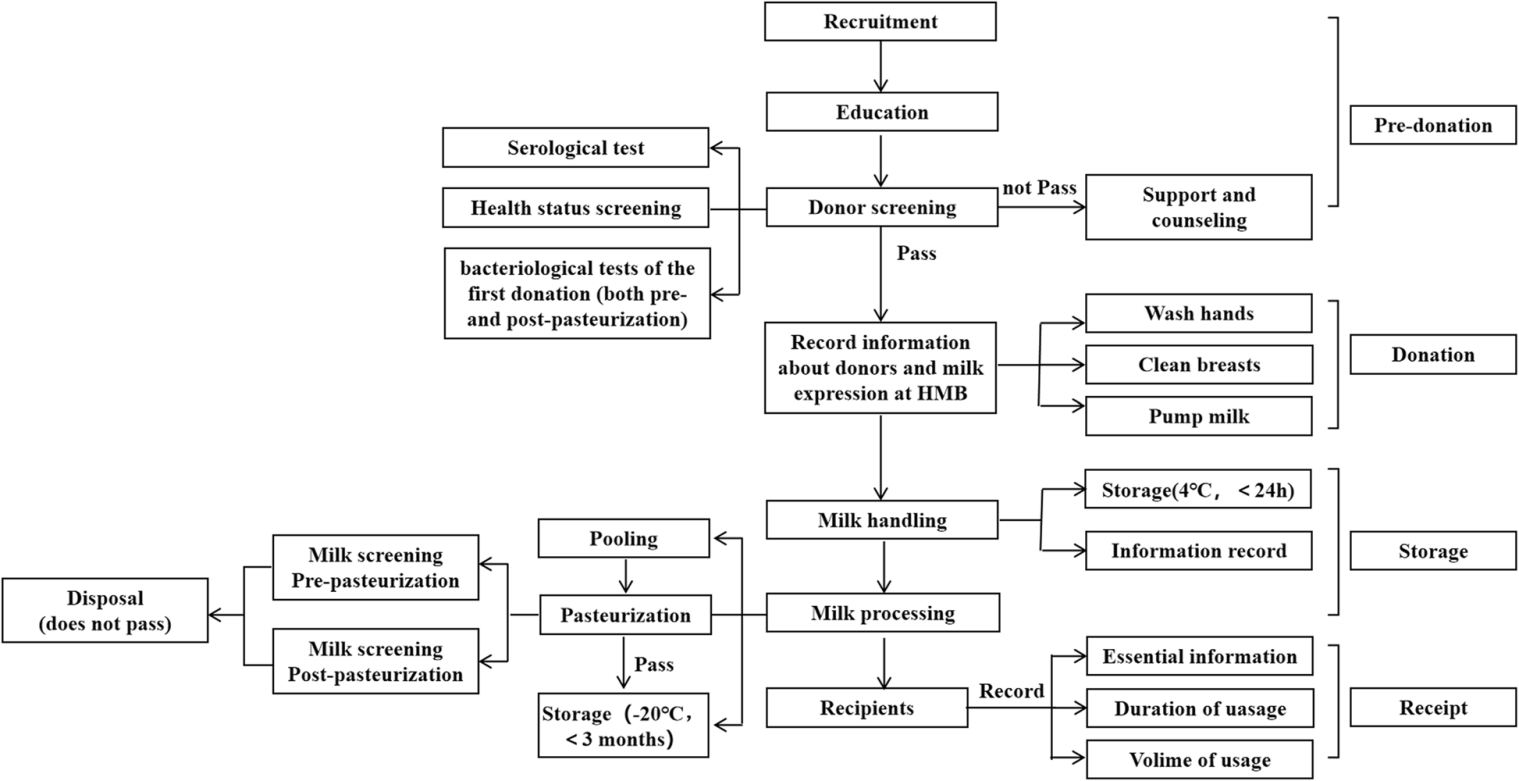
Current donation process at our HMB

All eligible donors receive on-site guidance by professionals and their essential information was recorded at the time of their first donation. Before breast milk collection, the donors are requested to wash their hands under strict instructions and clean their breasts with antiseptic wipes, especially around the nipple and areola. An electric milk pump is used, and all accessories are cleaned and disinfected before and after use. The collected breast milk is placed in a dedicated storage container and placed in a 4℃ refrigerator, and all the relevant information is recorded. All the donor milk is mixed and pasteurized (by continuous disinfection at 62.5 °C for 30 min) within 24 h after collection and then stored in a special -20℃ refrigerator for no more than 3 months. When the DHM was used, the information about recipient, duration and volume of usage is recorded (Fig. 1).

### Ethics

Our HMB was approved by the ethics committee of Maternity Hospital Affiliated to Nanjing Medical University before it was established. Potential donors are provided with the necessary information and sign the consent form before their first donation. Additionally, a medical informed consent form is signed by the parents of the recipients before DHM is provided. All the donors are anonymous and babies receive DHM from multiple donors.

## Results

### Infrastructures and cost analysis

Our HMB is equipped with an independent milk collection room, a hospital-level milk pump, a pasteurizer (temperature-controlled water bath), human milk storage containers, medical refrigerators (including a custom-made ordinary refrigerator and special ultra-low temperature refrigerator), computers with a supporting information management system, etc. The guidelines for organizational management; infrastructure; donor screening; and the collection, processing, storage, and provision of donated breast milk are in accordance with The Human Milk Banking Association of North America.

The total cost for 8 years was ¥1.94 million($306,051) for 7,396.5L of qualified DHM, mainly on testing and consumables. Both donation and provision of DHM are free. The average cost per liter of DHM was ¥262.3($41.4). Before the establishment of the HMB, we received a donation of ¥1 million from a group of good Samaritans, whom were parents of patients or private donations, as start-up capital. The rest of the expenses were borne by our neonate department, which covered 80% of the cost, and the hospital, which covered 20% of the cost. The annual cost with its breakdown is shown in Table 1.

**Table 1:** Costs of HMB activities using the activity-based costing model

Year	Consumable (¥)	Testing (¥)	Labor (¥)	Equipment (¥)	Total (¥)
2013.08–2013.12	25,015	38,298	20,000	57,258	140,571
2014	48,138.75	40,670	48,000	-	136,808.75
2015	47,415.25	67,012	48,000	-	162,427.25
2016	125,347.6	53,498	48,000	-	226,845.6
2017	245,467.5	36,285	66,000	-	347,752.5
2018	250,634.3	25,925	84,000	-	360,559.3
2019	178,741.4	44,645	84,000	-	307,386.4
2020	72,108.6	2,495	84,000	-	158,603.6
2021.01–07	48,894.5	1,525	49,000		99,419.5
Total	1,041,762.9	310,353	531,000	57,258	1,940,373.9

*HMB* Human milk bank, *RMB* Renminbi

### Characteristics of donors and donations

Between 1 August, 2013, and 31 July, 2021, a total of 1,805 mothers were enrolled, but 250(13.9%) did not pass the health screening or serological tests or their DHM did not pass the bacteriological test. The remaining 1,555(86.1%)qualified donors were mostly between 25 and 30 years of age (55.5%): 62.6% had a bachelor’s degree, 61.2% had given birth by vaginal delivery, 66.7% had had term births, and 80.1% were primipara (Table 2). Further, 46.2% of donors had donated milk more than 10 times (Table 2).

**Table 2:** Characteristics of donors

	***n*** (%)
Reasons for disqualification	
Failure to pass the physical examination	168 (67.2)
the appearance of donor milk is not well	17 (6.8)
Failure to pass the pre-pasteurization test	25 (10.0)
Failure to pass the post-pasteurization test	14 (5.6)
Others	26 (10.4)
Number of donations	
< 3	287 (18.5)
3–9	549 (35.3)
≥ 10	719 (46.2)
Preterm/term delivery	
Preterm	518 (33.3)
Term	1037 (66.7)
Time of first donation (postpartum)	
< 1 wk	153 (9.8)
1 wk to 1 mo	325 (20.9)
1–3 mo	789 (50.7)
3–6 mo	240 (15.4)
6–10 mo	48 (3.1)
Mode of delivery	
Vaginal delivery	951 (61.2)
Cesarean section	604 (38.8)
Number of children	
1	1245 (80.1)
≥ 2	310 (19.9)
Occupation	
Company employee	575 (37.0)
Government employee	197 (12.7)
Self-employed	205 (13.2)
Housewife	315 (20.2)
Others	263 (16.9)
Age (y)	
20–25	126 (8.1)
25–30	863 (55.5)
30–35	426 (27.4)
> 35	140 (9.0)
Education	
Higher than a bachelor’s degree	206 (13.2)
Bachelor’s degree	974 (62.6)
Junior college degree	134 (8.6)
Lower than a junior college degree	241 (15.5)

**Fig. 2:**
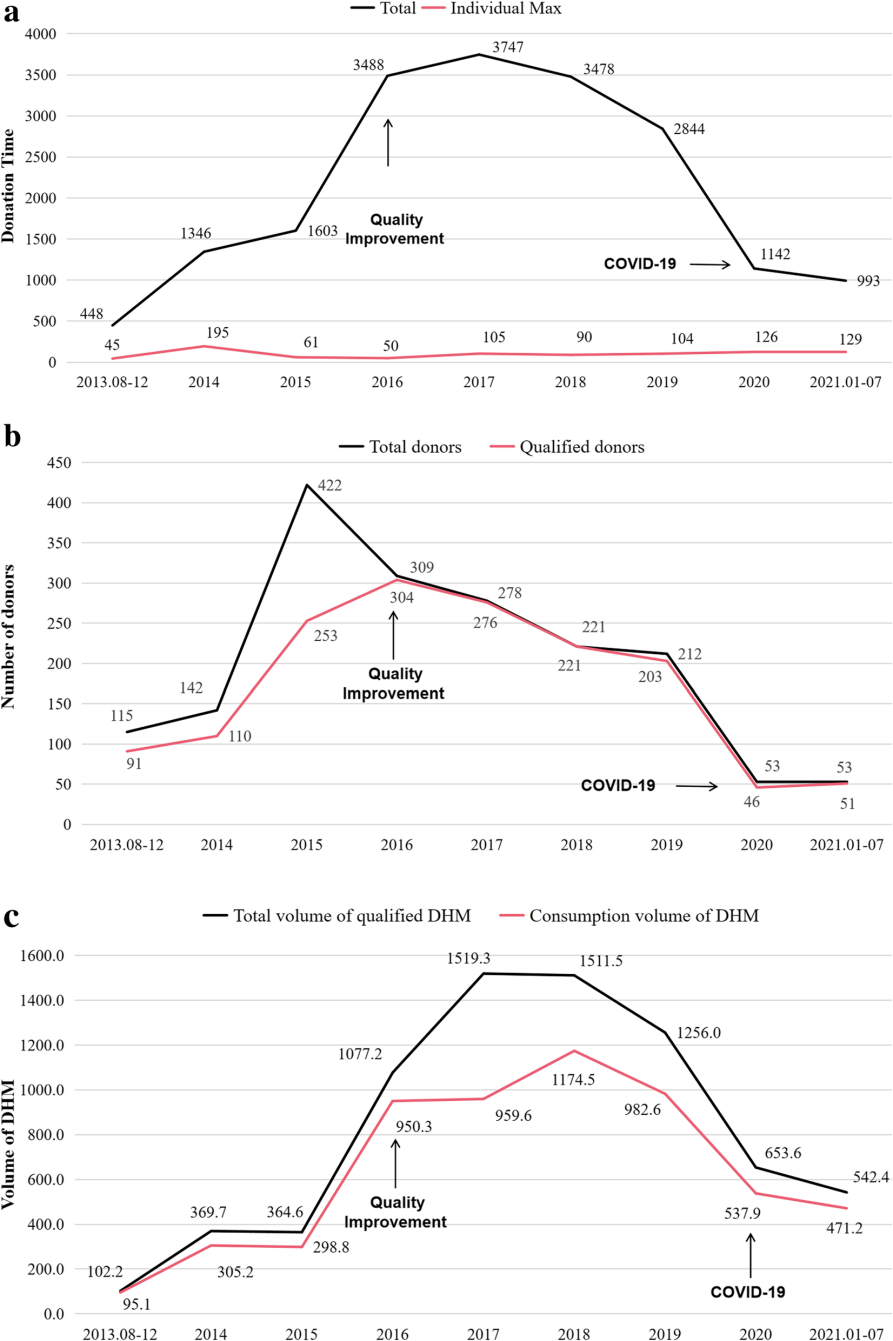
Characteristics of donors and donations. Number of donations (**A**), number of donors (**B**), volume of qualified DHM (**C**) over the 8-year period of operation of our HMB MOM: Mother’ own milk

The total number of donation times in 8 years was 19,089, and the annual maximum number of donation time for individual donor was 195 (Fig. 2A). In 2013, the number of qualified donors was 91 and the volume of qualified DHM was 102.2 L. As a result of continuous quality improvements, January 2016 [11] witnessed a significant increase in both the volume of qualified DHM(1519.3L) and the number of qualified donors(304). In 2020, the number of patients at our NICU decreased due to the COVID-19 pandemic, and the volume of qualified DHM(537.9L) and the number of qualified donors(46) consequentially decreased (Fig. 2B and C).

### Passing rate of bacteriological test

**Fig. 3:**
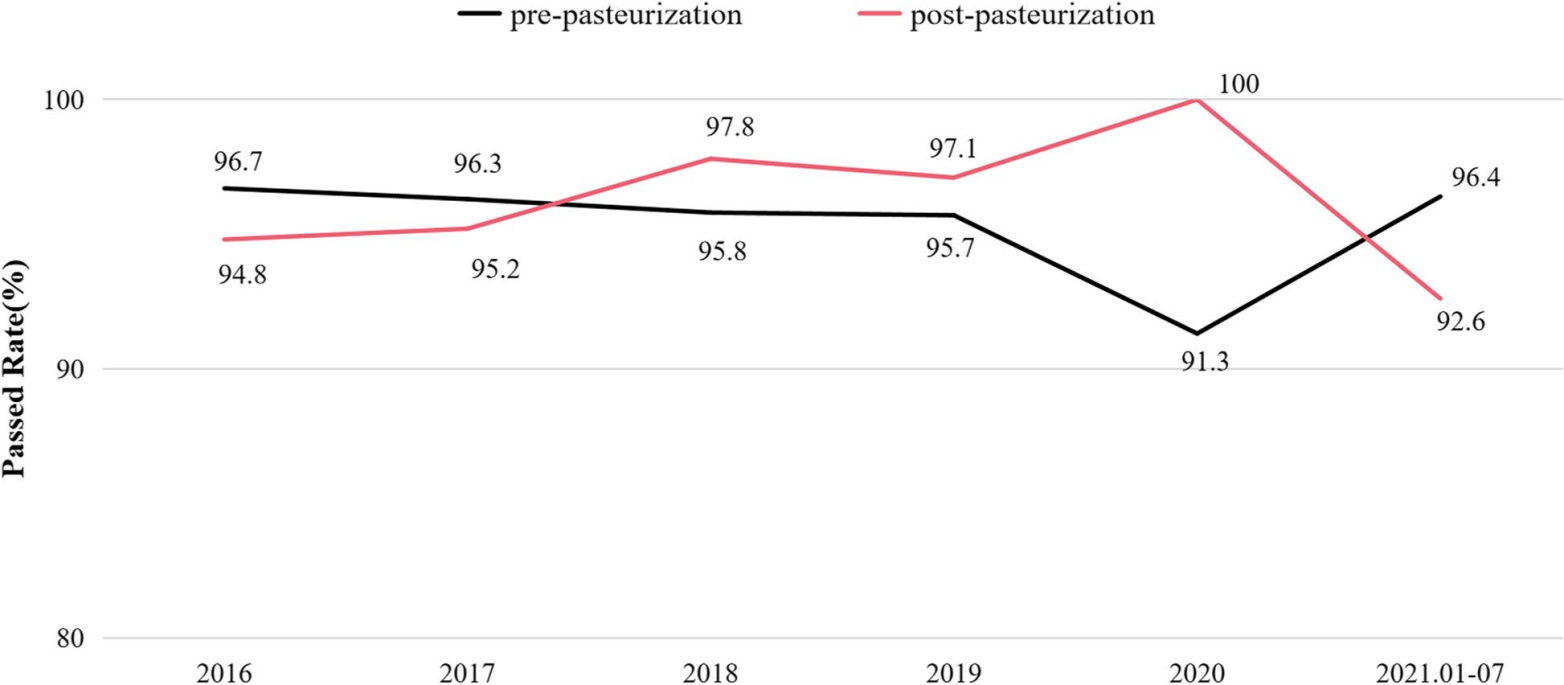
Proportion (%) of milk that passed the pre- and post-pasteurization bacteriological tests

Prior to January 2016, both pre- and post-pasteurization bacteriological test was conducted on qualified donors’ DHM only at the first time of donation. The data of these part was not recorded. From January 2016, on average, 95.4% of the batches passed the pre-pasteurization tests, and 96.3% passed the post-pasteurization tests (Fig. 3).

### Recipient characteristics

Over the 8-year study period, a total of 5,775.2 L of DHM was supplied to 9,207 infants (Fig. 2C), and the maximum individual volume was 13.7 L. Most of the recipients were preterm (83.3%), and 81.7% were single birth. In 64.4% of the recipients, the birth weight was between 1,500 g and 2,500 g, and in 41.7%, gestational age was between 34 to 37 wk. Further, 94.3% of them received DHM for less than 15 days, and the average of duration for receiving DHM was 4.5 days (Table 3).

**Table 3:** Characteristics of recipients

		***n*** (%)
Birth weight (g)		
< 1000		233 (2.5)
1000–1500		1398 (15.2)
1500–2500		5932 (64.4)
≥ 2500		1644 (17.9)
Gestational age (wk)		
< 28	326 (3.5)	
28–31		1179 (12.8)
31–34		2325 (25.3)
34–37		3842 (41.7)
≥ 37		1535 (16.7)
Time point at which DHM was started (postnatal)		
< 24 h		4254 (46.2)
24–48 h		3848 (41.8)
> 48 h		1105 (12.0)
Single/twins/triplet		
Single		7523 (81.7)
Twins		1664 (18.1)
≥ Triplets		20 (0.2)
Reasons for prescription		
Preterm		7417 (80.1)
Necrotizing enterocolitis		356 (3.9)
Severe infection or sepsis		718 (7.8)
Feeding intolerance		516 (5.6)
Duration for which DHM was provided		
< 15 d		8682 (94.3)
15 d to 1 mo		453 (4.9)
> 1 mo		72 (0.8)
Longest (d)		67
Average (d)		4.5

## Discussion

### Cost of the HMB

This study describes the operations of our HMB and analyzes the data gathered over the last 8 years of its existence. Over the 8-year period, the total expenditure of the HMB was 1.94 million RMB($306,051), including employee salary, materials costs, test costs, etc., and both the donation and provision of DHM were free. The average cost per liter of qualified DHM was ¥262($41.4). In comparison, studies from the United States have calculated that 1 L of DHM costs approximately US $150, and studies from Germany have reported a cost of $94 per liter of DHM [12–14]. The costs of our HMB are probably lower because most donors are not required to repeat the HBV,HCV,HIV and syphilis test when they donate within 6 months of their serological test, which is done during hospitalization at the time of delivery at our hospital. The cost of providing DHM is much higher than the cost of formula and mother’s own milk. However,breastfeeding could reduce the incidence of diseases, such as necrotizing enterocolitis and late onset sepsis, and save a lot of future medical costs [15]. Therefore, we also advocate and promote breastfeeding through various means, such as propaganda and education of breastfeeding to parents and medical staffs, etc., at our HMB.

### Characteristics of human milk donors

Over the 8-year period, a total of 250 mothers(13.9%) did not meet the criteria for donation because they did not pass the health screening or serological test or their DHM did passed the bacteriological tests (Table 1). In 2015, a total of 169 mothers did not qualify because the CMV-DNA test of their DHM was positive, and this phenomenon exists in most Chinese mothers [16]. In fact, after pasteurization or freeze, the CMV-DNA test shows negative results, which means that the donor milk is not likely to cause neonatal infection [17]. Thus, after the continuous quality improvements that were initiated in January 2016, we only used the donors’ serum CMV test(including IgG and IgM) for screening.

During the last 8 years of operation of the HMB, the number of qualified donors and the volume of qualified DHM first showed an increase and then a decrease. In 2016, we carried out quality improvement programs for the HMB, including conducting propaganda and education of breast milk feeding, establishing education sites of breast milk donation, maternal and child health publicity in shopping mall, improving the process of breast milk donation and processing, ect. [11]. These programs resulted in a considerable increase of the number of qualified donors(304) and the volume of qualified DHM(1519.3L).In the first three months of 2020, due to the restrictions policy for donors coming to the hospital, our HMB did not receive any DHM no matter collected at home or at our HMB.Then the epidemic eased and our HMB gradually returned to normal work. Therefor, the number of qualified donors(46) and the volume of qualified DHM(537.9L) decreased significantly in 2020.

Our donors were mainly aged between 25 and 30 years, and this is similar to the donor demographic of HMBs reported in Taiwan and Thailand [18, 19]. 33.3% of the donors in the present study delivered preterm infants. It is mainly because the mothers of preterm infants have a reduction in postpartum lactation due to physical reasons [20] and they did not have added breastmilk for donation. In the present study, 61.2% of donors gave birth by vaginal delivery. This percentage is different from that reported in Thailand [17], but is similar to a previous report in mainland China [7]. Further, 46.2% of the donors in this study donated more than 10 times, which is higher than the percentage reported in mainland China and indicates that we have a higher average number of donations than other domestic HMBs. This is probably attributable to the efforts of our staff in providing correct information and following up with donors. Additionally, 62.6% of the donors had a bachelor’s degree, which meant well-educated mothers tend to have better knowledge about and attitude towards milk donation [21]. Most of the donors began to donate milk at 1 month postpartum at the earliest, and the number of donors who started to donate deceased rapidly at 3–6 months postpartum. The main reason is probably that most women in China are only given 4–6 months of maternity leave. Once they resume work, their free time and volume of lactation probably decrease dramatically.

### Pasteurization

Although holder pasteurization (HoP,62.5°C for 30 min) may reduce the immunological components in donor milk, such as sCD14, however, this has little impact on the protein, fat, carbohydrates, some trace elements, and the activity of some enzymes [5, 22] that are very important and irreplaceable for the development of neonates, especially premature infants. Recently, a high-temperature short-time treatment(HTST, 72°C for 30s) was designed as an alternative for HMBs, which resulted in better preservation of the nutritional quality of DHM than HoP because relevant thermosensitive components (phospholipids, PUFAs, and BSSL) were less affected [23]. But we still choose HoP instead of HTST because the duration of HTST treatment, which had a greater influence on the nutrient composition of DHM than did the tested temperature, was harder to control [23].

### Recipients

The DHM in our HMB was only supplied to the neonates in our NICU and was not continued after discharge, and it was provided free of cost. This model is different from that described in previous reports from Thailand and the UK,which provide DHM for non-hospitalized infants [24, 25]. During the 8-year period, 9,207 newborns received DHM, most of whom were premature infants (80.1%), and the newborns had severe infection, feeding intolerance, and necrotizing enterocolitis. The maximum volume per neonate was 13.7 L, which was used by an extremely premature infant. The duration of use of DHM was mostly less than 15 days, and the average duration was 4.5 days, which is shorter than that previously reported in Scotland [26]. This is probably because most mothers have expressed enough breast milk to feed their own babies after a certain time point.

### Impact of COVID-19

During the COVID-19 pandemic, China implemented strict restriction policy, so we also added COVID-19 nucleic acid testing to the donor screening tests. Visitation was forbidden during newborn hospitalization, and patients from other provinces were not admitted to come to our hospital until recently. From February 4^th^ to March 4^th^ 2020, breast milk donation and transport to the hospital were not allowed in our hospital. These policies led to a significant reduction in both the number of donors and volume of DHM. Although this was accompanied by a significant reduction in the number of newborns as well as the demand for DHM in our hospital, due to pregnant women in other cities could not come to our hospital for childbirth, these conditions also led to the depletion of DHM stored at our HMB. However, it is worth noting that there was no case of COVID-19 infection at our hospital during this period.

### Conclusion

HMBs could support and promote breastfeeding, and provide better choices and better nutritional treatment options for children who cannot be breastfed by their mothers, especially premature infants. Additionally, it is very important in raising social awareness about the benefits of breastfeeding or DHM feeding, and actively publicize and provide breastfeeding guidance. Over the 8 years of operation of our HMB, through continuous quality improvement, our processes have been gradually finetuned and made efficient, and we will continue to provide DHM to newborns in the future, and provide some recommendations to other HMBs operating in China.
